# Age-specific humoral immune response to SARS-CoV-2: A comparative analysis of antibody levels in children and adults after vaccination with primary series or infection

**DOI:** 10.1371/journal.pone.0356178

**Published:** 2026-09-02

**Authors:** Brooke A. Hawkes, James Hollister, Cynthia Porter, Zoe L. Lyski, Jefferey L. Burgess, Karen Lutrick, Shawn C. Beitel, Ryan S. Sprissler, Katherine D. Ellingson

**Affiliations:** 1 Epidemiology and Biostatistics Department, Mel and Enid Zuckerman College of Public Health, University of Arizona, Tucson, Arizona, United States of America; 2 Department of Immunobiology, University of Arizona, Tucson, Arizona, United States of America; 3 Department of Community, Environment and Policy, Mel and Enid Zuckerman College of Public Health, University of Arizona, Tucson, Arizona, United States of America; 4 Family and Community Medicine, College of Medicine – Tucson, University of Arizona, Tucson, Arizona, United States of America; 5 University of Arizona Genetics Core, Office for Research, Innovation and Impact, University of Arizona, Tucson, Arizona, United States of America; UniCamillus: Saint Camillus International University of Health and Medical Sciences, ITALY

## Abstract

**Background:**

Age is a crucial factor in COVID-19 risk and severity, however its role in humoral immunity is unclear. This study compares associations between age and immune response following COVID-19 vaccination and SARS-CoV-2 infection.

**Methods:**

Data from the Arizona Healthcare, Emergency Response, and Other Essential Workers study (AZ-HEROES), a prospective cohort, were used to create two analytic groups: 1) a post-vaccination cohort including individuals who received two doses of the monovalent ancestral mRNA COVID-19 vaccine without prior SARS-CoV-2 infection; and 2) a post-infection cohort including unvaccinated individuals after primary infection. The analysis included data from September 2020 to April 2023. Children (<18 years) and adults (18 + years) were compared using linear regression, with immune response measured using log-transformed area under the curve (AUC) for receptor-binding domain (RBD) and spike protein subunit (S2). Finer age groups (<12, 12−17, 18−50, 50+) were also described. Adjusted models controlled for chronic conditions, medications, days since vaccination or infection, recent exposure to SARS-CoV-2 (post-vaccination only), strain type (post-infection only), and sociodemographic variables.

**Results:**

The post-vaccination analysis included 749 participants, while the post-infection analysis included 203 participants. Participants were predominantly non-Hispanic White adults, with median ages of 43 and 40 years in the post-vaccination and post-infection cohorts, respectively. Most reported no chronic conditions. In adjusted post-vaccination analysis, adults had 7% higher RBD AUC (95% CI: 1.01–1.13) and 20% higher S2 AUC (95% CI: 1.13, 1.28) than children, although these results were attenuated when restricted to individuals taking the BNT162b2 vaccine only. Post-infection, adults had 22% higher S2 AUC (95% CI: 1.05, 1.40), while RBD differences were not significant. By finer age breakdown, adolescents (12−17 years) had stronger responses than children younger than 12 post-vaccination, with 26% higher RBD AUC (95% CI: 1.13–1.40) and 29% higher S2 AUC (95% CI: 1.14–1.46).

**Conclusions:**

COVID-19 immune responses differ between children and adults, with adolescents exhibiting stronger immune response to vaccination than children younger than 12, however vaccine dosage may play a role in this observed difference. Age-related differences after infection were limited to S2 antibody responses.

## Introduction

The manifestation of Coronavirus Disease 2019 (COVID-19) varies significantly between adults and children, with children generally exhibiting milder symptoms and better prognoses compared to adults [1–3]. For most infectious diseases, the most vulnerable age groups include the very young and the very old, however COVID-19 has not reflected this typical pattern. Although approximately 6% of the United States population is made up of children 5 years and younger, fewer than 0.1% of COVID-19 deaths are from this population, even though more than 90% of children under 4 have tested positive for previous or current infection by SARS-CoV-2 [4].

The effect that age has on the SARS-CoV-2 specific antibody response to infection and vaccination is variable across the literature. Some studies showed elevated pediatric response to vaccination [5–7] and infection [3,6,8–10] compared to adults. Others reported elevated antibody responses in adults to vaccination [11] and infection [12,13]. There are also studies showing a comparable response in children and adults, both post-vaccination [5,14] and post-infection [14,15]. One study reported 37.0% of children with positive SARS-CoV-2 PCR results demonstrated immunoglobulin G (IgG) seroconversion, compared to 76.2% of adults in the study [12]. However, a separate study reported significantly higher and more persistent SARS-CoV-2-specific IgG and neutralization activity over a 6-month period in children (6 months-18 years old) compared to adults (18−54 years old) [6]. Existing research is often limited in sample size, making these differences even harder to distinguish. These mixed findings indicate a need for more robust estimates of immune response in larger cohorts to clarify how age affects SARS-CoV-2 immune response. Understanding the key differences in immune response by age could help to guide future vaccination and public health strategies. While age-related differences in COVID-19 severity are well established, how humoral immune responses to SARS-CoV-2 infection or vaccination differ by age remains unclear.

The Arizona Healthcare, Emergency Response, and Other Essential Workers Study (AZ HEROES) was a prospective cohort that actively surveilled front line workers for SARS-CoV-2 infection and immunologic response to infection and vaccination. Frontline workers included those who could not socially distance during the peak of the COVID-19 pandemic due to their jobs, including health care workers, first responders, food service workers, childcare workers, and more [16]. This cohort was used to compare SARS-CoV-2 specific antibody responses in children and adults, as well as more granular categories to further explore the role of age. Our overall objective was to examine antibody responses following completion of primary mRNA COVID-19 vaccine series and SARS-CoV-2 infection in unvaccinated individuals. Here, we aimed to contribute to the understanding of how age influences SARS-CoV-2 immune responses. We hypothesized that SARS-CoV-2 humoral immune responses following vaccination and infection would differ across age groups.

## Methods

### Study design

For this study, two cohorts were nested within the AZ HEROES prospective cohort study. This included a cohort made up of frontline workers 18 years and older, and a cohort of children aged 6 months to 17 years. Frontline worker enrollment and data collection began in July 2020, and incorporated children in July 2021. Both cohorts were followed longitudinally to assess SARS-CoV-2 infection and COVID-19 vaccination status.

Across both cohorts, baseline surveys were completed at enrollment to capture sociodemographic information, social behaviors, health insurance status, medical history (including underlying medical conditions, daily medications, and vaccine history), and history of SARS-CoV-2 infection. Participants (or parents/guardians) were asked whether they had tested positive for SARS-CoV-2 prior to enrollment and, if so, to provide the date or approximate date of their positive test. COVID-19 vaccination status was collected at enrollment or when participants became eligible to receive the vaccine and was updated through periodic follow-up surveys. Vaccination data was verified via vaccination cards.

Participants from both cohorts provided a mid-turbinate nasal specimen weekly and at the onset of COVID-19-like illness symptoms throughout followup. Specimens were tested for SARS-CoV-2 at the Marshfield Clinic Laboratory (Marshfield, WI) using real time reverse transcription-polymerase chain reaction (rRT-PCR). Data collection ended April 2023.

### Adult cohort

Beginning in July 2020, frontline workers in Arizona were enrolled and followed as part of the AZ HEROES Study [17]. Eligible adult participants were individuals working at least 20 hours per week in occupations requiring frequent direct contact with individuals outside their household during the COVID-19 pandemic. In addition to shared baseline measures, adult participants provided occupational characteristics.

### Child cohort

In July 2021, the HEROES network was expanded to include children aged 6 months to 17 years. Informed consent was obtained from parents or legal guardians, and children aged 7–17 years provided assent to participate.

### Ethical approval

All study protocols were approved by the University of Arizona Institutional Review Board (IRB) (protocol #2006729444), and informed consent was obtained from all participants.

### Data collection

#### Blood collection.

All enrolled adult participants had the option to provide a blood sample at enrollment, following SARS-CoV-2 infection, after receiving any COVID-19 vaccine, every 11–13 weeks, and at the end of the study. Children had the option to provide a blood sample at enrollment, following SARS-CoV-2 infection, after receiving any COVID-19 vaccine, and at the end of the study. Children providing a blood draw required consent from a parent or guardian. To capture peak antibody response following vaccination or infection, this analysis was limited to participants who provided blood samples 14–60 days after completing the primary vaccine series or after a first-time SARS-CoV-2 infection. At each time point, 5–10 mL of whole blood was collected and processed according to CDC guidelines for serum collection [18]. Samples were frozen, transported on dry ice, and used for the serological assays detailed below [18].

### Enzyme-linked immunosorbent assay (ELISA)

All serum samples were sent to the University of Arizona Genetics Core laboratory for semiquantitative ELISA testing against SARS-CoV-2 Washington-1 (WA1) receptor-binding domain (RBD) and S2, following established protocols [19,20]. Five threefold dilutions of sera were made starting at a 1:60 dilution and ending at 1:4860. An area under the serial dilution curve (AUC) was calculated using the optical density values at each dilution. AUC values represent a weighted sum of the optical density value at each dilution [21]. Whole genome sequencing was conducted on a subset of samples against Omicron (BA.2) RBD if eligible (cycle threshold value <30) [22]. Both RBD and S2 antibody can measure an individual’s immune response to a SARS-CoV-2 infection or COVID-19 vaccination, however the RBD test specifically detects antibodies that block the virus from attaching to cells, whereas the S2 test detects antibodies targeting the structural region responsible for fusing the virus into the cell [23,24]. Optical density was measured at five 3-fold dilutions starting at 1:60, and antibody levels were quantified as the area under the serial dilution curve (AUC). The AUC method is widely recognized for summarizing semiquantitative ELISA results, offering improved coverage probabilities of titration curves compared to other metrics, such as end-point titer [19,25,26].

### Inclusion and exclusion criteria

The post-vaccination analysis for this study included data collected from December 16, 2020 to September 7, 2022, as this encompassed the timeframe in which vaccinations were available and a sample of individuals still had not had a primary SARS-CoV-2 infection. Participants were included in the post-vaccination analysis if they had received at least two doses of the monovalent BNT162b2 vaccine or the mRNA-1273 vaccine and had a blood draw 14–60 days after vaccination. They were excluded from the analytic sample set if they had been previously infected with SARS-CoV-2, received a non-mRNA COVID-19 vaccine, received a bivalent or non-ancestral COVID-19 vaccine, or were missing covariate data.

The post-infection analysis included data collected from September 24, 2020 to April 14, 2023, the dates that included unvaccinated participants with a first-time infection. Participants were included in the post-infection analysis if they had a first-time SARS-CoV-2 infection during the study period and a blood draw 14–60 days afterwards. They were excluded from the analytic sample if they had received a COVID-19 vaccine prior to blood collection or were missing covariate data. A supplementary analysis of vaccinated individuals was conducted for only those vaccinated with monovalent BNT162b2.

### Statistical methods

#### Outcomes of interest.

The primary outcomes of interest were the AUC values for RBD and S2 antibodies obtained from ELISA assays for both the post-infection and post-vaccination cohorts. The AUC values were natural log-transformed prior to analysis [25]. Exponentiated beta coefficients from linear regression models were then reported, representing the geometric mean ratio (GMR). In this context, the GMR quantifies the proportional change in AUC values for RBD and S2 antibodies post-infection or vaccination.

#### Exposure of interest.

The primary exposure of interest was age group, defined as children younger than 18 years old and adults 18 years and older. To further examine age and antibody response, we also broke down age groups further: children less than 12 years, adolescents 12–17 years, adults 18–50 years, and adults older than 50 years. The age of children was broken down by difference in vaccine dosage, [27] while adults were divided based on cut-offs defined in the literature as important in terms of immune system decline among working-age adults [28].

#### Covariates.

For all adjusted analyses, covariates of interest were determined a priori based on existing literature [6,29]. They included sex, city of participant’s address, household size, race and ethnicity, body mass index (BMI) category, chronic conditions (binary variable, yes/no), daily medication use, and days since event. Chronic diseases included asthma, chronic lung disease, cancer, diabetes, heart disease, hypertension, immunosuppression, kidney disease, liver disease, neurologic or neuromuscular disease or disorder, and autoimmune disease. These conditions were identified using questionnaire data indicating whether participants had received medical care for any of these conditions within the past 12 months. Household size was included as a covariate due to its potential to influence exposure intensity and, therefore, viral load [30,31]. Vaccine manufacturer was excluded as a covariate due to sparse data among children, but a supplementary analysis limited to just those who received BNT162b2 was conducted to assess potential differences in antibody response by manufacturer.

For post-infection individuals, SARS-CoV-2 variant was also considered. Variant of infection was confirmed by whole-genome sequencing for eligible specimens or estimated by using the state-specific predominant variant at the time of infection according to Centers for Disease Control and Prevention data. For analysis, strain was categorized as “Omicron” and “Other.”

Due to limited data availability, race and ethnicity were grouped into a binary variable (“Non-Hispanic White” and “Other”), and BMI categories were simplified into “Underweight/Normal Weight” and “Overweight/Obese.” Covariates were selected a priori and assessed for collinearity. For children, BMI-for-age percentiles were used to define BMI categories [32]. These variables were included as potential confounders of the association between age and antibody response and were not evaluated as primary exposures of interest.

### Demographics by age group

The association between demographics and age group were examined in the two cohorts, post-infection and post-vaccination. These were assessed using *χ* [2], Fisher exact, and *t* tests. Differences were considered statistically significant at *p* < 0.05.

### Age group and antibody response

RBD and S2 were first compared by age and infection status (infection or vaccination) to identify potential differences. Unadjusted linear regression models and models fully adjusted for the covariates above were used to examine the relationship between age group and immune markers in two groups: vaccinated individuals and those previously infected with SARS-CoV-2. Since AUC values were transformed for analysis, exponentiated beta coefficients were reported, representing the geometric mean ratio. A sensitivity analysis restricted to BNT162b2 recipients was conducted to evaluate whether associations between age and antibody response were consistent when all participants received the same vaccine. All analyses were conducted using SAS OnDemand for Academics, R version 4.3.3, and GraphPad Prism software (version 9.4.1 for Mac; GraphPad Software). BMI for age percentile in children were calculated using the R package “cdcanthro” [33].

## Results

### Baseline demographic characteristics: Post-SARS-CoV-2 vaccination participants

**Fig 1:**
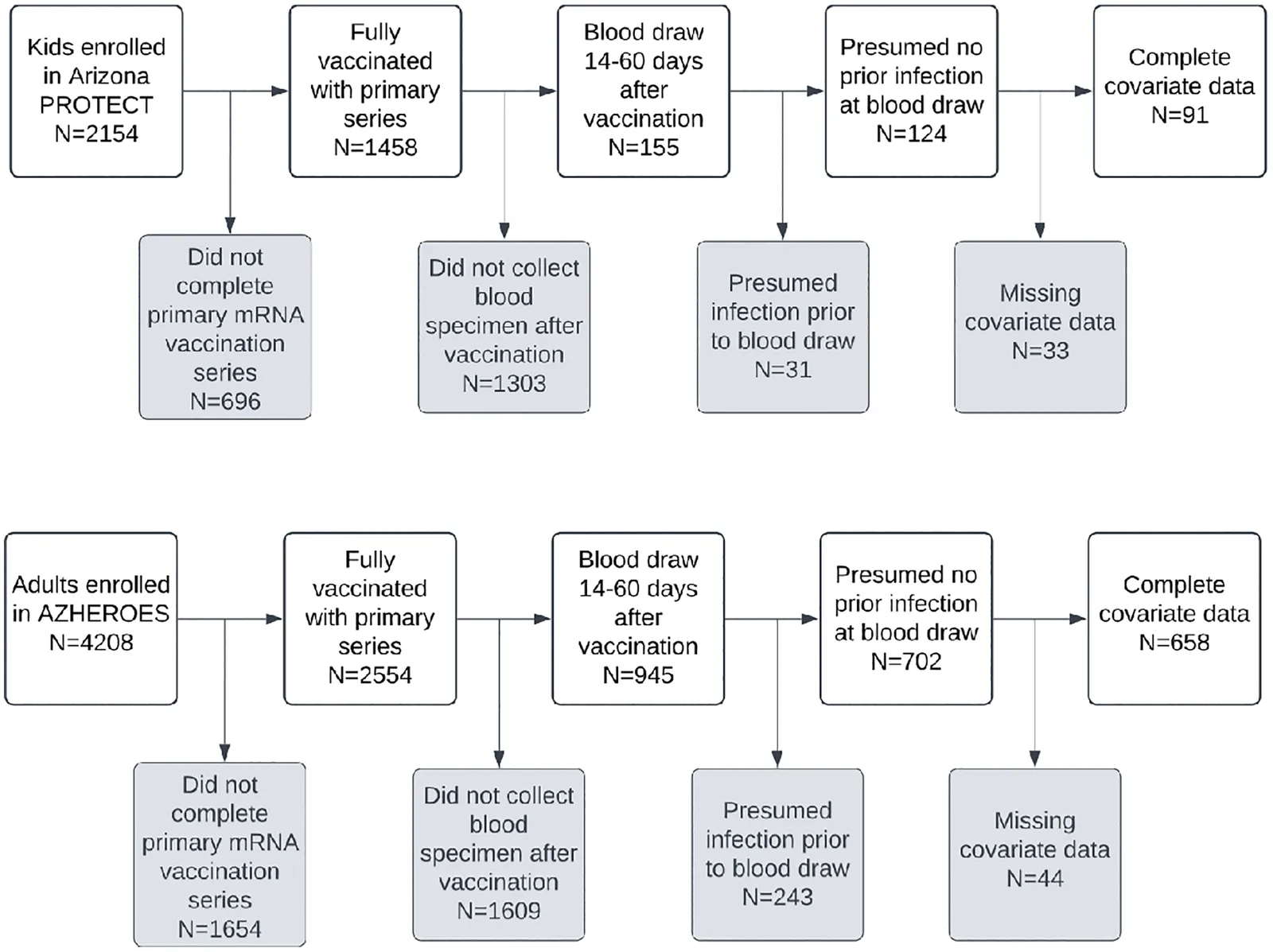
Vaccination Analysis Exclusion Criteria. This figure outlines the number of individuals eliminated from the AZHEROES cohort before the post-vaccination analysis.

After the exclusion criteria were applied, 749 participants remained in the post-vaccination primary analysis (Fig 1). The sample included 91 children and 658 adults and was predominantly female (64.1%), non-Hispanic White (75.9%), located in Tucson (61.2%), and without chronic conditions (73.5%). All children received the BNT162b2 vaccine series, whereas adults received either BNT162b2 (63.8%) or mRNA-1273 (36.2%).

Several participant characteristics differed significantly between children and adults. As expected, adults had a higher prevalence of chronic conditions, daily medication use, and overweight/obesity than children. They were also more likely to be female, have a smaller house size, have less days since vaccination. Location also differed between children and adults, with children more likely to be from Tucson or Phoenix, compared to other places in Arizona. Race and ethnicity distributions were similar between groups. See Table 1 for details.

**Table 1:** Baseline Demographic Characteristics of Participants of the HEROES Cohort: September 2020 to April 2023 (n = 952).

	Post-COVID-19 Vaccination			Post-SARS-CoV-2 Infection		
Characteristic	Total n (%*) (N = 749)	Children (<18 years) n (%†) (N=91)	Adults (18 + years) n (%†) (N=658)	Total n (%*) (N = 203)	Children (<18 years) n (%†) (N=60)	Adults (18 + years) n (%†) (N=143)
Sex‡						
Male	268 (35.7)	47 (17.5)	221 (82.5)	108 (53.2)	37 (34.3)	71 (65.7)
Female	481 (64.1)	44 (9.1)	437 (90.9)	95 (46.8)	23 (24.2)	72 (75.8)
Race and Ethnicity¶						
Non-Hispanic White	569 (75.9)	69 (12.1)	500 (87.9)	145 (71.4)	44 (30.3)	101 (69.7)
Other	180 (24.0)	22 (12.2)	158 (87.8)	58 (28.6)	16 (27.6)	42 (72.4)
Location†						
Tucson	449 (61.2)	68 (14.8)	391 (85.2)	117 (57.6)	31 (26.5)	86 (73.5)
Phoenix	182 (24.3)	15 (8.2)	167 (91.8)	53 (26.1)	17 (32.1)	36 (67.9)
Other places in AZ	108 (14.4)	8 (7.4)	100 (92.6)	33 (16.3)	12 (36.4)	21 (63.6)
Household size, mean (SD) ‡§	3.27 (1.5)	4.5 (1.3)	3.1 (1.5)	3.71 (1.7)	3.34 (1.7)	4.62 (1.5)
Chronic conditions‡§						
Yes, any	198 (26.4)	11 (5.6)	187 (94.4)	51 (25.1)	8 (15.7)	43 (84.3)
None	551 (73.5)	80 (14.5)	471 (85.5)	152 (74.9)	52 (34.2)	100 (65.8)
Takes >= 1 daily medication‡§#						
Yes	371 (49.5)	19 (5.1)	352 (94.9)	82 (40.4)	9 (11.0)	73 (89.0)
No	378 (50.4)	72 (19.0)	306 (81.0)	121 (59.6)	51 (42.1)	70 (57.9)
SARS-CoV-2 variant‡§						
Omicron	n/a	n/a	n/a	58 (28.6)	25 (43.1)	33 (56.9)
Other#	n/a	n/a	n/a	145 (71.4)	35 (24.1)	110 (75.9)
COVID-19 vaccine manufacturer						
BNT 162b2	511 (68.1)	91 (17.8)	420 (82.2)	n/a	n/a	n/a
mRNA-1273	238 (31.7)	0 (0.0)	238 (100.0)	n/a	n/a	n/a
Days since vaccination, mean (SD)‡	25.0 (10.1)	33.4 (12.4)	23.8 (9.1)	n/a	n/a	n/a
BMI Category‡§**						
Underweight/ Normal Weight	348 (46.4)	74 (21.3)	274 (78.7)	94 (46.3)	51 (54.3)	43 (45.7)
Overweight/ Obese	401 (53.5)	17 (4.2)	384 (95.8)	109 (53.7)	9 (8.3)	100 (91.7)

*Column %

†Row %

‡Significantly different between post-vaccination children and adults. Assessed using χ2, Fisher exact, and t tests, considered statistically significant at p < 0.05.

§Significantly different between post-infection children and adults. Assessed using χ2, Fisher exact, and t tests, considered statistically significant at p < 0.05.

¶Self-reported

#Answered “yes” to survey question “Do you take a daily medication prescribed by a doctor or healthcare professional?”

**Body mass index (BMI) for adults: weight(kg)/height(m) [2] (under/over BMI of 25). BMI for children: CDC’s BMI-for-age percentiles (under/over 85% percentile).

### Baseline demographic characteristics: Post-SARS-CoV-2 infection participants

**Fig 2:**
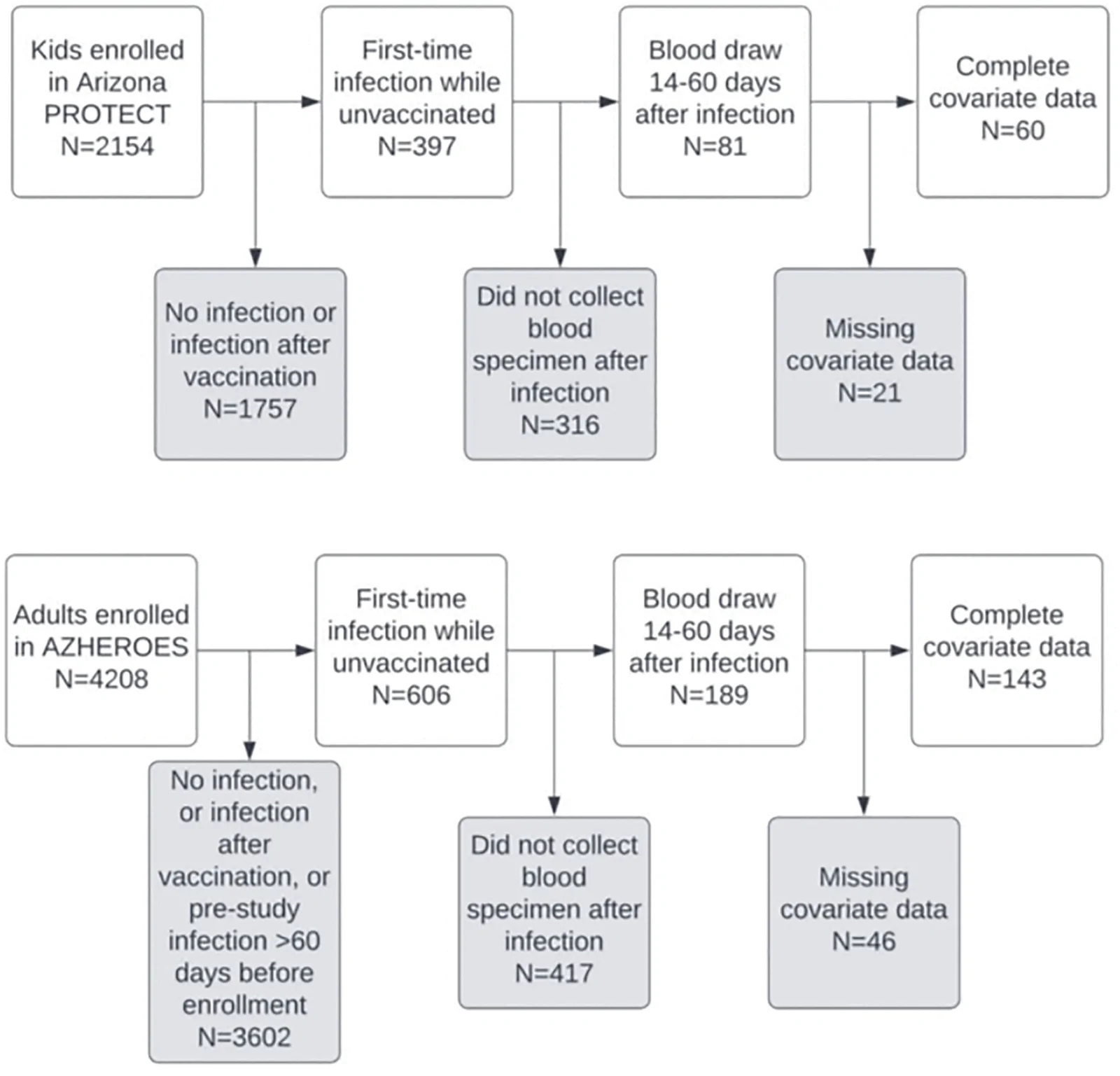
Infection Analysis Exclusion Criteria. This figure outlines the number of individuals eliminated from the AZHEROES cohort before the post-infection analysis.

For post-infection participants, 203 individuals were included in the primary analysis after exclusion criteria were applied (Fig 2). This sample was mostly non-Hispanic white (71.4%) from Tucson (57.6%), without chronic conditions (74.9%), not taking daily medications (59.6%), and infected with a non-Omicron variant (71.4%). There were 60 children and 143 adults in this sample. Complete summary statistics for post-infection participants are shown in Table 1. See Supplementary Table 2 for a further breakdown by age.

Adults had a higher prevalence of chronic conditions (84.3% vs 15.7%), daily medication use (89.0% vs 11.0%), and overweight/obesity (91.7% vs 8.3%) compared with children. In contrast, children lived in larger households on average (mean = 4.6 vs 3.3 people). Sex and location distributions were similar between groups (Table 1).

### Antibody response by age group: Stratified by infection history

Post-infection and post-vaccination RBD AUC values differed significantly, while S2 AUC values did not. RBD AUC values, irrespective of age, were higher in vaccinated individuals compared to infected (P = 0.0007). However, S2 AUC values were not significantly different (slope P = 0.01, elevation P = 0.83). See S1 Fig for more information.

### Antibody Response by Age Group: Post-Vaccination

Before adjusting for confounding variables, adults had a significantly higher antibody response compared to children following vaccination (Table 2). Specifically, RBD AUC was 12% higher in adults (95% CI: 1.07–1.18), while S2 AUC was 32% higher (95% CI: 1.24–1.40).

**Table 2:** Unadjusted and Adjusted Linear Regression Models of Age Group and Antibody Response (Log-transformed RBD and S2 AUC) Post-COVID-19 Vaccination or SARS-CoV-2 Infection in Children (<18) and Adults (18+). September 2020 to April 2023.

Response by Age Group (years)	Unadjusted Model		Adjusted Model*		Partially Adjusted Model†	
	Exponentiated β (95% CI) ‡	*p*-value §	Exponentiated β (95% CI)	*p*-value	Exponentiated β (95% CI)	*p*-value
Post-Vaccination RBD AUC						
<18	1.00 (ref)	--	1.00 (ref)	--	1.00 (ref)	--
18+	1.12 (1.07, 1.18)	<0.01	1.07 (1.01, 1.13)	0.02	1.08 (1.03, 1.14)	<0.01
Post-Vaccination S2 AUC						
<18	1.00 (ref)	--	1.00 (ref)	--	1.00 (ref)	--
18+	1.32 (1.24, 1.40)	<0.01	1.20 (1.13, 1.28)	<0.01	1.21 (1.14, 1.29)	<0.01
Post-Infection RBD AUC						
<18	1.00 (ref)	--	1.00 (ref)	--	1.00 (ref)	--
18+	1.61 (1.27, 2.04)	<0.01	1.21 (0.95, 1.54)	0.12	1.29 (1.05, 1.58)	0.02
Post-Infection S2 AUC						
<18	1.00 (ref)	--	1.00 (ref)	--	1.00 (ref)	--
18+	1.27 (1.11, 1.46)	<0.01	1.22 (1.05, 1.40)	0.01	1.24 (1.09, 1.42)	<0.01

Abbreviations: AUC, area under the serial dilution curve; CI, confidence interval; RBD, receptor-binding domain; β, beta coefficient

Reference group is children <18 years old in all models

*Adjusted for sex, city of participant’s address, household size, ethnicity, BMI category, chronic conditions, daily medication, days since event, recent contact with a COVID-positive individual (*post-vaccination only*), SARS-CoV-2 strain (*post-infection only*)

† Adjusted only for covariates with p < 0.05 (*see appendix for details)*

‡Exponentiated beta coefficients (resulting in the geometric means ratios) and confidence intervals to account for log-transformation of AUC values in the model.

§Significant at p < 0.05

After adjusting for potential confounders, post-vaccination, RBD AUC was 7% higher in adults (95% CI: 1.01–1.13) and S2 AUC was 20% higher (95% CI: 1.13–1.28). Findings were generally similar in the sensitivity analysis restricted to BNT162b2 recipients, although effect estimates were attenuated (Supplementary Table 1).

When analyzed with narrower age categories, further differences in humoral immune response differences by age were revealed (Table 3). In post-vaccination individuals, children less than 12 years of age consistently had the lowest immune response, both in unadjusted and adjusted RBD and S2 AUC models. Compared to children younger than 12, RBD AUC values were 26%, 14%, and 5% high after adjustment in adolescents (12−17 years), adults (18−50 years), and older adults (50 + years), respectively. However, these differences were non-significant among older adults (95% CIs: 1.13–1.40, 1.07–1.21, 0.98–1.12). S2 AUC values varied slightly, with values 29%, 25%, and 22% higher after adjustment in adolescents, adults, and older adults, respectively, compared to children younger than 12 (95% CIs: 1.14–1.46, 1.17–1.35, 1.13–1.32).

**Table 3:** Unadjusted and Adjusted Linear Regression Models of Age Group and Antibody Response (Log-transformed RBD and S2 AUC) Post-COVID-19 Vaccination or SARS-CoV-2 Infection in Children <12, Adolescents (12-17), Adults (18-50) and Older Adults (50+). September 2020 to April 2023.

Response by Age Group (years)	Unadjusted Model		Adjusted Model*		Partially Adjusted Model†	
	Exponentiated β (95% CI) ‡	*p*-value §	Exponentiated β 95% CI)	*p*-value	Exponentiated β (95% CI)	*p*-value
Post-Vaccination RBD AUC						
< 12	1.00 (ref)	--	1.00 (ref)	--	1.00 (ref)	--
12-17	1.21 (1.09, 1.35)	<0.01	1.26 (1.13, 1.40)	<0.01	1.26 (1.14, 1.40)	<0.01
18-50	1.21 (1.15, 1.29)	<0.01	1.14 (1.07, 1.21)	<0.01	1.15 (1.08, 1.22)	<0.01
50+	1.12 (1.06, 1.19)	<0.01	1.05 (0.98, 1.12)	0.18	1.05 (0.99, 1.12)	0.11
Post-Vaccination S2 AUC						
< 12	1.00 (ref)	--	1.00 (ref)	--	1.00 (ref)	--
12-17	1.23 (1.08, 1.40)	<0.01	1.29 (1.14, 1.46)	<0.01	1.29 (1.14, 1.46)	<0.01
18-50	1.40 (1.30, 1.50)	<0.01	1.25 (1.17, 1.35)	<0.01	1.27 (1.18, 1.36)	<0.01
50+	1.37 (1.27, 1.47)	<0.01	1.22 (1.13, 1.32)	<0.01	1.24 (1.15, 1.33)	<0.01
Post-Infection RBD AUC						
< 12	1.00 (ref)	--	1.00 (ref)	--	1.00 (ref)	--
12-17	0.86 (0.51, 1.43)	0.55	0.83 (0.53, 1.29)	0.40	0.87 (0.56, 1.35)	0.52
18-50	1.52 (1.16, 2.00)	<0.01	1.11 (0.84, 1.47)	0.48	1.25 (0.99, 1.59)	0.06
50+	1.64 (1.20, 2.24)	<0.01	1.08 (0.78, 1.49)	0.65	1.27 (0.96, 1.68)	0.09
Post-Infection S2 AUC						
< 12	1.00 (ref)	--	1.00 (ref)	--	1.00 (ref)	--
12-17	1.03 (0.76, 1.39)	0.85	1.00 (0.76, 1.31)	1.00	1.03	0.82
18-50	1.30 (1.11, 1.53)	<0.01	1.21 (1.02, 1.44)	0.03	1.20	0.02
50+	1.24 (1.03, 1.48)	0.02	1.07 (0.88, 1.31)	0.49	1.08	0.39

Abbreviations: AUC, area under the serial dilution curve; CI, confidence interval; OR, odds ratio; RBD, receptor-binding domain; β, beta coefficient

Vaccination group sample sizes: <12 (n=70), 12-17 (n=22), 18-50 (n=403), 50+ (n=255)

Infection group sample sizes: <12 (n=49), 12-17 (n=11), 18-50 (n=93), 50+ (n=50)

Reference group is children <12 years old in all models

*Adjusted for sex, city of participant’s address, household size, ethnicity, BMI category, chronic conditions, daily medication, days since event, SARS-CoV-2 strain (*post-infection only*)

† Adjusted only for covariates with p < 0.05 (*see appendix for details)*

‡Exponentiated beta coefficients (resulting in the geometric means ratios) and confidence intervals to account for log-transformation of AUC values in the model

§Significant at p < 0.05

### Antibody Response by Age Group: Post-Infection

We found that adults had a significantly higher antibody response compared to children younger than 18 following infection in an unadjusted analysis (Table 2). Post-infection, RBD was 61% higher in adults (95% CI: 1.27–2.04) and S2 AUC was 27% higher (95% CI: 1.11–1.46). After adjustment, RBD AUC was 21% higher, although not significantly different, in adults (95% CI: 0.95–1.54) and S2 AUC was 22% higher (95% CI: 1.05–1.40).

The response observed in post-infection individuals varied slightly when breaking down age categories further. For example, after adjustment children younger than 12 and adolescents had similar RBD AUC (95% CI: 0.53–1.29), while adults had an RBD AUC 11% higher than children younger than 12 (95% CI: 0.84–1.47) and older adults 8% higher (95% CI: 0.78–1.49). None of these differences were statistically significant. After adjustment, S2 responses compared to children younger than 12 were similar to adolescents (95% CI 0.76–1.31), and older adults (95% CI: 0.88–1.31). Similarly, although these differences were observed, none were statistically significant. However, the highest response was seen in adults, 21% higher than children younger than 12 (95% CI 1.02–1.44).

## Discussion

This study suggests that age is an important factor in SARS-CoV-2 antibody responses. Notably, both vaccination and infection yielded a higher response in adults compared to children, although this difference was limited to the S2 antibody resonse post-infection. RBD and S2 AUC values were significantly higher in adults before and after adjusting for potential confounders. However, once age was broken down into subcategories, the relationship was not as clear. There was more variation in response to vaccination compared to infection, although this is consistent with age-specific vaccine dosing schedules.

In June 2022, the Centers for Disease Control and Prevention (CDC) had recommended COVID-19 vaccination for all individuals over 6 months of age [34]. For individuals who are not moderately or severely immunocompromised, the BNT162b2 vaccine (Pfizer-BioNTech) was recommended at the following dosages: 3 µg for ages 6 months through 4 years, 10 µg for ages 5 years to 11 years, and 30 µg for ages 12 years and older [27]. For the mRNA-1273 vaccine (Moderna), the following dosages were recommended: 25 µg for ages 6 months through 11 years and 50 µg for ages 12 years and older [27].

Children younger than 12 consistently had the lowest immune response post-vaccination, followed by older adults, younger adults, and then adolescents. It is interesting to note the difference between children younger than 12 and adolescents, considering both age groups made up the category of children in our primary analysis. Because vaccine dosage varies systematically by age in pediatric populations, the observed differences between children younger than 12 years and adolescents may reflect age-related immunologic differences, vaccine dosage differences, or a combination of both. Our study design does not allow these effects to be separated. At age 12, individuals receive the same dose as adults (30 µg) compared to children aged 6 months through 4 years who receive 3 µg and children 5–11 years who receive 10 µg. Post-infection, children younger than 12 and adolescents had the lowest immune response, followed by older adults and then younger adults. Reduced antibody titers observed in older adults is consistent with the existing literature that establishes strong evidence in the loss of immune competence with age [35,36]. The similarity seen between children younger than 12 and adolescents post-infection that is not observed post-vaccination could be in part due to differences in age-specific vaccine dosing schedules. Regardless, it is interesting to note that while adolescents demonstrated the highest immune response post-vaccination, their response post-infection was one of the lowest. These findings suggest that age-related differences in humoral immune responses are nuanced and may differ across stages of development. The particularly strong responses observed among adolescents following vaccination highlight the importance of considering age-specific immune responses when evaluating vaccine performance and designing future immunization strategies.

Although these findings are consistent with past studies, [6,8–10,12–14] observing a difference between children and adult SARS-CoV-2 immune responses, most of the prior studies observed a higher immune response in children, contrasting this study. Many previously conducted studies have relatively low sample sizes (between 25 and 125 participants), so it is possible that some of the differences observed were due to lack of statistical power. However, of the two studies identified that had comparable samples to this study (669 participants and 842 participants), both indicated higher immune response in children [6,7].It is difficult to make direct comparisons between these studies, however, due to variations in study design. For example, one looked at immune response to COVID-19 vaccination using IgG titers, and did not eliminate or differentiate analysis based on if an individual had a SARS-CoV-2 infection prior to vaccination [7]. Considering that hybrid immunity usually results in much higher antibody titers than vaccination only, it is not surprising that results were inconsistent [37]. The other study was able to break down age group categories further, including age groups less than 5, 5–11, and 12–17 years [6]. They also did not have participants older than 54 years old included in the study. Timing of specimen collection is also an important factor to consider; this study looked at IgG response one month and 6 months post-infection or vaccination, whereas our data did not extend further than 60 days post-infection or vaccination. At the one-month time point, there was no significant difference in spike-specific IgG levels between age groups following infection. However, spike-specific IgG levels after vaccination were significantly higher in children aged less than 5 years old compared to children 12–17 years old and adults. Because our study measured ELISA-derived RBD and S2 area-under-the-curve values rather than spike-specific IgG titers, and because our analyses were limited to samples collected within 60 days of infection or vaccination, direct comparisons should be interpreted cautiously. Due to data availability, we also could not look at children less than 5 years old as an individual group.

This study has several unique strengths. To date, most studies comparing SARS-CoV-2 immune response in adults and children have low sample sizes. Our larger sample size allowed us to compare these two age groups with greater statistical power and break down our age groups into more granular categories to explore the nuance in these differences. The frequent collection of participant serum samples allowed us to specifically look at responses within 14–60 days of an event (either vaccination or infection), which allowed for consistency when comparing immune responses. This study also self-confirmational assays interrogating antibodies against two separate domains of the spike protein to further enhance our findings. Finally, following enrollment in AZ-HEROES, participants underwent weekly SARS-CoV-2 PCR testing and frequent vaccination surveys. This rigorous monitoring ensured that blood draws included in the analysis were unlikely to violate exclusion criteria, such as those occurring after a repeat SARS-CoV-2 infection or an additional COVID-19 vaccine.

Several limitations should be considered. First, immune responses were only evaluated within 60 days of infection or vaccination, excluding the assessment of age-related differences over time. Although participants with known prior SARS-CoV-2 infection were excluded from the post-vaccination analysis, asymptomatic infections may have resulted in exposure misclassification since pre-vaccination serologic screening was not performed. We also did not evaluate hybrid immunity. Additionally, ELISA measured binding antibodies but did not assess neutralizing activity or T-cell responses. Although the overall sample size was large, relatively few children, especially those younger than 12 years, and fewer post-infection participants limited statistical power for subgroup analyses. Residual confounding is also possible, as not all comorbidities and potential confounders could be accounted for. Selection bias may have occurred because blood collection was voluntary, particularly among children, and the cohort consisted primarily of healthy individuals with mild infections. Finally, because the study was conducted in Arizona, the findings may not be generalizable to other populations.

The primary purpose of this study was to evaluate differences in antibody responses to SARS-CoV-2 infection and vaccination by age. Humoral antibody levels have been shown to be a crucial factor in SARS-CoV-2 infection and reinfection, so understanding what factors effect these levels is crucial. We highlighted differences that exist between children and adults but also highlighted how the age groups among children may be of especially high importance. Understanding immune responses post-vaccination is beneficial for providing well-supported vaccine recommendations for different age groups. Additionally, providing details of immune response to the SARS-CoV-2 infection to the public will help with more precise COVID-19 messaging. Much of the COVID-19 pandemic brought confusion among the public due to several unknowns. An added layer of understanding immune response is an important step in having a more complete understanding of the disease and therefore being able to communicate that understanding with the public in a precise way. Future research should focus on more granular age categories, especially in children. Immunity changes over time and hybrid immunity should be evaluated to be more reflective of the general population today.

## Supporting information

S1 Fig Differences in Antibody AUC values by Age. Antibody AUC values were compared between adults and children against RBD and S2 of WA-1 spike. P values are based on Mann-Whitney non-parametric test.
(TIF)

## References

[1] Morrell ED, Mikacenic C. Differences between Children and Adults with COVID-19: It’s Right under Our Nose. Am J Respir Cell Mol Biol. 2022;66(2):122–3. doi: 10.1165/rcmb.2021-0455ED 34758269 PMC8845138

[2] Dhochak N, Singhal T, Kabra SK, Lodha R. Pathophysiology of COVID-19: Why Children Fare Better than Adults?. Indian J Pediatr. 2020;87(7):537–46. doi: 10.1007/s12098-020-03322-y 32410003 PMC7221011

[3] Mentor G, Farrar DS, Di Chiara C, Dufour M-SK, Valois S, Taillefer S, et al. The Effect of Age and Comorbidities: Children vs. Adults in Their Response to SARS-CoV-2 Infection. Viruses. 2024;16(5):801. doi: 10.3390/v16050801 38793682 PMC11126068

[4] CDC. Surveillance and Data Analytics. Covid. https://www.cdc.gov/covid/php/surveillance/index.html. 2026. Accessed 2026 June 29.

[5] Nziza N, Deng Y, Wood L, Dhanoa N, Dulit-Greenberg N, Chen T, et al. Humoral profiles of toddlers and young children following SARS-CoV-2 mRNA vaccination. Nat Commun. 2024;15(1):905. doi: 10.1038/s41467-024-45181-7 38291080 PMC10827750

[6] Kim M, Cheng WA, Congrave-Wilson Z, Marentes Ruiz CJ, Turner L, Mendieta S, et al. Comparisons of Pediatric and Adult SARS-CoV-2-Specific Antibodies up to 6 Months after Infection, Vaccination, or Hybrid Immunity. J Pediatric Infect Dis Soc. 2024;13(1):91–9. doi: 10.1093/jpids/piad107 38016076 PMC10824260

[7] Bohn MK, Steele S, Adeli K. SARS-CoV-2 serology in pediatrics: Seroprevalence studies in unvaccinated children and humoral antibody response post vaccination. Clin Biochem. 2023;119:110630. doi: 10.1016/j.clinbiochem.2023.110630 37549823

[8] Stoddard CI, Sung K, Yaffe ZA, Weight H, Beaudoin-Bussières G, Galloway J, et al. Elevated binding and functional antibody responses to SARS-CoV-2 in infants versus mothers. Nat Commun. 2023;14(1):4864. doi: 10.1038/s41467-023-40554-w 37567924 PMC10421871

[9] Long-term immune response to SARS-CoV-2 infection among children and adults after mild infection. https://pubmed.ncbi.nlm.nih.gov/35816313/ Accessed 2024 March 14. 10.1001/jamanetworkopen.2022.21616 PMC9280400 35816313

[10] Yang HS, Costa V, Racine-Brzostek SE, Acker KP, Yee J, Chen Z, et al. Association of Age With SARS-CoV-2 Antibody Response. JAMA Netw Open. 2021;4(3):e214302. doi: 10.1001/jamanetworkopen.2021.4302 33749770 PMC7985726

[11] Functional T cell response to COVID-19 vaccination with or without natural infection with SARS-CoV-2 in adults and children. Scientific Reports. 10.1038/s41598-025-95870-6 PMC12006499 40247005

[12] Toh ZQ, Anderson J, Mazarakis N, Neeland M, Higgins RA, Rautenbacher K, et al. Comparison of Seroconversion in Children and Adults With Mild COVID-19. JAMA Netw Open. 2022;5(3):e221313. doi: 10.1001/jamanetworkopen.2022.1313 35262717 PMC8908077

[13] Weisberg SP, Connors TJ, Zhu Y, Baldwin MR, Lin W-H, Wontakal S, et al. Distinct antibody responses to SARS-CoV-2 in children and adults across the COVID-19 clinical spectrum. Nat Immunol. 2021;22(1):25–31. doi: 10.1038/s41590-020-00826-9 33154590 PMC8136619

[14] Pillay A, Yeola A, Tea F, Denkova M, Houston S, Burrell R, et al. Infection and Vaccine Induced Spike Antibody Responses Against SARS-CoV-2 Variants of Concern in COVID-19-Naïve Children and Adults. J Clin Immunol. 2023;43(8):1706–23. doi: 10.1007/s10875-023-01540-5 37405544 PMC10661752

[15] Bartsch YC, Wang C, Zohar T, Fischinger S, Atyeo C, Burke JS, et al. Humoral signatures of protective and pathological SARS-CoV-2 infection in children. Nat Med. 2021;27(3):454–62. doi: 10.1038/s41591-021-01263-3 33589825 PMC8315827

[16] Join the AZ HEROES Study | AZHEROES | Healthcare, Emergency Response, and Other Essential Workers Surveillance. https://azheroes.arizona.edu/join-az-heroes. Accessed 2026 June 29.

[17] Lutrick K, Ellingson KD, Baccam Z, et al. COVID-19 infection, reinfection, and vaccine effectiveness in a prospective cohort of Arizona frontline/essential workers: The AZ HEROES research protocol. JMIR Res Protoc. 2021;10(6):e28925. doi: 10.2196/28925 PMC8386365 34057904

[18] CDC. Interim Guidelines for Collecting and Handling of Clinical Specimens for COVID-19 Testing. https://www.cdc.gov/covid/hcp/clinical-care/clinical-specimen-guidelines.html. 2024. Accessed 2024 November 3.

[19] Shroff RT, Chalasani P, Wei R, Pennington D, Quirk G, Schoenle MV, et al. Immune responses to two and three doses of the BNT162b2 mRNA vaccine in adults with solid tumors. Nat Med. 2021;27(11):2002–11. doi: 10.1038/s41591-021-01542-z 34594036 PMC9004706

[20] Ripperger TJ, Uhrlaub JL, Watanabe M, Wong R, Castaneda Y, Pizzato HA, et al. Orthogonal SARS-CoV-2 Serological Assays Enable Surveillance of Low-Prevalence Communities and Reveal Durable Humoral Immunity. Immunity. 2020;53(5):925-933.e4. doi: 10.1016/j.immuni.2020.10.004 33129373 PMC7554472

[21] Yu X, Gilbert PB, Hioe CE, Zolla-Pazner S, Self SG. Statistical approaches to analyzing HIV-1 neutralizing antibody assay data. Stat Biopharm Res. 2012;4(1):1–13. doi: 10.1080/19466315.2011.633860 24660049 PMC3959164

[22] Mak J, Khan S, Britton A, et al. Association of messenger RNA coronavirus disease 2019 (COVID-19) vaccination and reductions in post COVID conditions following severe acute respiratory syndrome coronavirus 2 infection in a US prospective cohort of essential workers. J Infect Dis. 2025;231(3):665–76. doi: 10.1093/infdis/jiae556 39531735 PMC11913564

[23] Premkumar L, Segovia-Chumbez B, Jadi R, Martinez DR, Raut R, Markmann A, et al. The receptor binding domain of the viral spike protein is an immunodominant and highly specific target of antibodies in SARS-CoV-2 patients. Sci Immunol. 2020;5(48):eabc8413. doi: 10.1126/sciimmunol.abc8413 32527802 PMC7292505

[24] Guenthoer J, Garrett ME, Lilly M, Depierreux DM, Ruiz F, Chi M, et al. The S2 subunit of spike encodes diverse targets for functional antibody responses to SARS-CoV-2. PLoS Pathog. 2024;20(8):e1012383. doi: 10.1371/journal.ppat.1012383 39093891 PMC11324185

[25] Amanat F, Stadlbauer D, Strohmeier S, Nguyen THO, Chromikova V, McMahon M, et al. A serological assay to detect SARS-CoV-2 seroconversion in humans. Nat Med. 2020;26(7):1033–6. doi: 10.1038/s41591-020-0913-5 32398876 PMC8183627

[26] Yu X, Gilbert PB, Hioe CE, Zolla-Pazner S, Self SG. Statistical approaches to analyzing HIV-1 neutralizing antibody assay data. Stat Biopharm Res. 2012;4(1):1–13. doi: 10.1080/19466315.2011.633860 24660049 PMC3959164

[27] Centers for Disease Control and Prevention. 2024–2025 COVID-19 vaccine immunization schedule for people 6 months of age and older. Published September 19, 2024. Accessed August 27, 2026. https://restoredcdc.org/www.cdc.gov/vaccines/covid-19/downloads/COVID-19-immunization-schedule-ages-6months-older.pdf

[28] Weyand CM, Goronzy JJ. Aging of the immune system. Mechanisms and therapeutic targets. Ann Am Thorac Soc. 2016;13(Suppl 5):S422–8. doi: 10.1513/AnnalsATS.201602-095AW PMC5291468 28005419

[29] Lyski ZL, Porter C, Uhrlaub JL. Humoral immune response to messenger RNA coronavirus disease 2019 vaccination among children aged 5–11 years in a multisite prospective cohort study, September 2021–September 2022. Open Forum Infectious Diseases. 2023;10(8):ofad431. doi: 10.1093/ofid/ofad431 PMC10468733 37663086

[30] Cerami C, Popkin-Hall ZR, Rapp T. Household transmission of severe acute respiratory syndrome coronavirus 2 in the United States: living density, viral load, and disproportionate impact on communities of color. Clin Infect Dis. 2022;74(10):1776–85. doi: 10.1093/cid/ciab701 34383889 PMC8436395

[31] Baldi ME, Marsou Z, Chellat FZ, Benjelloun MC, Belahsen MF, Houssaini TS, et al. Household transmission of SARS-CoV-2 during the first wave of the COVID-19 pandemic: an analysis of secondary attack rates in Moroccan households. BMC Infect Dis. 2025;25(1):1022. doi: 10.1186/s12879-025-11464-7 40813964 PMC12355784

[32] CDC Extended BMI-for-Age Growth Charts for Children and Adolescents With Very High BMIs. https://www.cdc.gov/nccdphp/dnpao/growthcharts/extended-growth-charts.html. Accessed 2024 August 23.

[33] CDC. R Programs for Various Growth Charts. Growth Chart Training. https://www.cdc.gov/growth-chart-training/hcp/computer-programs/r-programs.html. 2024. Accessed 2025 January 16.

[34] Centers for Disease Control and Prevention. Coronavirus Disease 2019. https://www.cdc.gov/media/releases/2022/s0618-children-vaccine.html. 2022. Accessed 2025 January 16.

[35] Weng N-P. Aging of the immune system: how much can the adaptive immune system adapt?. Immunity. 2006;24(5):495–9. doi: 10.1016/j.immuni.2006.05.001 16713964 PMC2266981

[36] Goyani P, Christodoulou R, Vassiliou E. Immunosenescence: Aging and Immune System Decline. Vaccines. 2024;12(12):1314. doi: 10.3390/vaccines12121314 39771976 PMC11680340

[37] Ntziora F, Kostaki EG, Karapanou A, Mylona M, Tseti I, Sipsas NV, et al. Protection of vaccination versus hybrid immunity against infection with COVID-19 Omicron variants among Health-Care Workers. Vaccine. 2022;40(50):7195–200. doi: 10.1016/j.vaccine.2022.09.042 36150972 PMC9482842

